# Editorial to the Special Issue “Retinopathies: A Challenge for Early Diagnosis, Innovative Treatments, and Reliable Follow-Up”

**DOI:** 10.3390/medicina61040662

**Published:** 2025-04-03

**Authors:** Dario Rusciano, Stefania Marsili

**Affiliations:** 1 Fidia Ophthalmics, 95125 Catania, Italy; 2 Georgia Institute of Technology, Atlanta, GA 30332, USA

The Special Issue “Retinopathies: A Challenge for Early Diagnosis, Innovative Treatments, and Reliable Follow-Up” brings together a diverse yet interconnected collection of research papers that collectively address the multifaceted challenges of retinal diseases. These contributions, spanning clinical presentations, surgical outcomes, innovative therapies, and cutting-edge diagnostic tools, form a unified narrative of progress in understanding and treating retinopathies. By integrating genetic insights, therapeutic innovations, and advanced technologies such as artificial intelligence (AI) and optical coherence tomography (OCT), this Special Issue highlights the transformative potential of multidisciplinary approaches in ophthalmology.

At the heart of this Special Issue is the recognition that retinal diseases, despite their diverse manifestations, share common challenges that necessitate a unified approach to management. Conditions such as diabetic retinopathy, age-related macular degeneration (AMD), and retinal vein occlusions exemplify how early diagnosis is crucial. For instance, the ETDRS report n.9 [1] first highlighted that early detection of diabetic retinopathy through regular screening can decrease the risk of vision loss by up to 95%. Similarly, advancements in optical coherence tomography (OCT) have allowed for earlier identification of AMD, as demonstrated by Schmidt-Erfurth et al. [2], who found that timely intervention can substantially slow disease progression and preserve vision. Following diagnosis, innovative treatment modalities play a pivotal role in managing these conditions. The introduction of anti-vascular endothelial growth factor (anti-VEGF) therapies has revolutionized the treatment of neovascular AMD, providing significant improvements in visual acuity. However, the efficacy of these therapies hinges on timely administration; delays in treatment initiation can lead to irreversible vision loss [3]. Furthermore, establishing reliable follow-up care is essential for monitoring treatment efficacy and disease progression. A study by Yannuzzi et al. [4] highlighted that patients with diabetic retinopathy who adhered to regular follow-up appointments had significantly better visual outcomes compared to those who missed follow-ups, stressing the importance of consistent monitoring in preventing blindness.

Thus, this Special Issue emphasizes the need for interdisciplinary collaboration and continued research aimed at overcoming these shared obstacles. By focusing on early diagnosis, innovative therapies, and dependable follow-up care, we can advance clinical practice and improve patient-centered outcomes in the field of ophthalmology.

Pediatric rhegmatogenous retinal detachment (RRD), for instance, is a rare but complex condition, often complicated by delayed diagnosis and surgical challenges. Unlike adult RRD, which is frequently associated with age-related vitreous degeneration or trauma, pediatric cases are more likely to stem from congenital anomalies (e.g., Stickler syndrome or retinopathy of prematurity), high myopia, or prior ocular surgery. The rarity of the condition in children contributes to delayed diagnosis, as symptoms (e.g., floaters, photopsia, or vision loss) may be overlooked or misinterpreted—particularly in young patients who cannot articulate their complaints. Additionally, the surgical challenges are significant: pediatric vitreoretinal anatomy (e.g., stronger vitreoretinal adhesions, smaller ocular dimensions) and a higher propensity for postoperative proliferation (e.g., proliferative vitreoretinopathy, PVR) complicate repair. Long-term visual outcomes often depend on prompt intervention, but even with successful reattachment, amblyopia and strabismus may further compromise functional recovery [5]. Thus, pediatric RRD demands a high index of suspicion, multidisciplinary management, and tailored surgical approaches to optimize outcomes. Alabbasi et al. [6] provide a comprehensive retrospective analysis of 89 eyes from 70 children, revealing a high prevalence of prior intraocular surgeries (31.5%) and associated ocular diseases (65.2%). Despite achieving a remarkable 98.9% anatomical success rate, the study underscores the variability in visual outcomes, with 35% of eyes reaching only hand motion vision or worse. This emphasizes the critical need for early detection and intervention, setting the stage for improved clinical protocols and future research.

Similarly, age-related macular degeneration (AMD), a leading cause of blindness [7], presents significant challenges, particularly in its dry form, which accounts for most cases but lacks curative treatments. Unlike wet AMD, which can be managed with anti-VEGF injections, dry AMD progresses slowly, often remaining unnoticed until advanced geographic atrophy (GA) develops. Although recent FDA-approved therapies (e.g., pegcetacoplan, avacincaptad pegol) aim to slow GA progression, they cannot restore vision and offer only modest benefits [8]. The disease’s complex mechanisms—including oxidative stress, inflammation, and RPE degeneration—hinder treatment development, leaving a high unmet need for effective therapies [9]. Current research explores complement inhibitors, stem cells, and neuroprotection, but no breakthrough solutions yet exist. Anitua et al. [10] explore the potential of plasma rich in growth factors (PRGF) in a mouse model of AMD, demonstrating its protective effects against retinal pigment epithelium (RPE) degeneration and photoreceptor loss. These findings open new avenues for growth factor-based therapies, offering hope for halting or even reversing the progression of dry AMD. Complementing this, Brinkmann et al. [11] investigate Faricimab, a bispecific antibody targeting both VEGF and angiopoietin-2 (Ang2), in neovascular AMD. Using OCT angiography (OCTA), they reveal significant changes in choroidal flow signals, suggesting that dual-pathway inhibition may enhance therapeutic efficacy. Together, these studies highlight the potential of innovative therapies to address the unmet needs in AMD treatment.

Genetic insights are revolutionizing our understanding of retinal diseases, uncovering key mutations and molecular pathways that drive conditions like retinitis pigmentosa, Stargardt disease, and age-related macular degeneration (AMD). Advances in genomic sequencing and gene-editing technologies, such as CRISPR, have enabled researchers to identify disease-causing variants (e.g., in ABCA4 for Stargardt or CFH for AMD) and develop targeted therapies, including the first FDA-approved gene therapy for inherited retinal disease (voretigene neparvovec for RPE65 mutations). These discoveries not only improve diagnostic precision but also pave the way for personalized treatments, offering hope for previously untreatable genetic blindness [12,13]. Ongoing research into gene therapy, antisense oligonucleotides, and stem cell-based approaches continues to expand therapeutic possibilities for retinal disorders. D’Esposito et al. [14] identify a prevalent nonsense variant in the RP1 gene (p.Ser740 *) associated with autosomal dominant retinitis pigmentosa (ADRP) in western Sicily, suggesting a founder effect in the region. This discovery not only enhances our understanding of the genetic basis of ADRP but also paves the way for targeted gene therapies, such as gene editing or translational read-through approaches. Piergentili et al. [15] further contribute to this genetic narrative by describing an atypical late-onset RP phenotype linked to a specific CRX gene mutation (p.Tyr142Cys) in Italian patients. These findings underscore the importance of genetic testing in diagnosing atypical cases and tailoring personalized treatment strategies.

In the realm of pediatric retinal diseases, retinoblastoma remains a significant challenge as the most common primary intraocular malignancy in children. This aggressive cancer, caused by mutations in the RB1 tumor suppressor gene, requires early detection to prevent metastasis and vision loss. While advancements in chemotherapy (intra-arterial and intravitreal), laser therapy, and targeted treatments have improved survival rates in high-income countries to over 95%, significant disparities persist in low-resource settings where late-stage diagnoses remain common [16]. Emerging therapies, such as RB1-targeted gene therapy [17], offer promise, but managing advanced cases still often necessitates enucleation. The disease also presents unique diagnostic dilemmas, as its initial symptoms (leukocoria or strabismus) can mimic benign conditions, underscoring the need for increased awareness and accessible screening programs worldwide. Dogan et al. [18] investigate the potential of a combination of boswellic acid (BA) and cisplatin, demonstrating its ability to modulate NF-κB signaling, reduce inflammation, and induce apoptosis in retinoblastoma cells. This study adds to the growing body of evidence supporting the use of natural compounds as adjunctive therapies in cancer treatment, highlighting the need for further research into their molecular mechanisms.

The systemic impact of modifiable risk factors, particularly smoking, on retinal health represents a critical and preventable dimension of ocular disease. Smoking has been strongly linked to the development and progression of age-related macular degeneration (AMD), with studies demonstrating a 2–4 fold increased risk among smokers due to oxidative stress, vascular endothelial dysfunction, and inflammation [19]. Beyond AMD, smoking contributes to retinal vein occlusions, diabetic retinopathy progression, and toxic optic neuropathy through mechanisms involving hypoxia, increased blood viscosity, and direct cytotoxic effects on retinal ganglion cells [20]. Importantly, smoking cessation has been shown to reduce these risks significantly [21]. These findings underscore the importance of public health initiatives targeting smoking cessation as part of comprehensive retinal disease prevention strategies, while also highlighting the need for clinician awareness and patient education regarding this modifiable risk factor. Nebbioso et al. [22] use OCTA, spirometry, and thrombus formation analysis to reveal significant alterations in macular vascular density and pulmonary function in smokers. These findings highlight the interconnectedness of systemic and ocular health, emphasizing the importance of addressing smoking as a preventable risk factor in retinal diseases.

Advancements in diagnostic technologies, particularly artificial intelligence (AI) and optical coherence tomography (OCT), are revolutionizing retinal disease management by enabling earlier, more precise detection and monitoring. AI algorithms, trained on vast datasets of retinal images, can now identify subtle pathological features of conditions like diabetic retinopathy, age-related macular degeneration (AMD), and glaucoma with accuracy rivaling human experts [23]. Meanwhile, ultra-high-resolution OCT and OCT angiography (OCTA) provide unprecedented visualization of retinal microstructures and vasculature, facilitating the detection of subclinical changes before symptom onset [24]. Together, AI and OCT are shifting paradigms toward proactive, personalized retinal care, although challenges remain in standardization, real-world implementation, and equitable access to these innovations. Parmar et al. [25] provide a comprehensive overview of AI applications in retinal disease diagnosis, emphasizing its potential to enhance screening efficiency and facilitate early detection. While AI offers transformative possibilities, challenges such as the “black box phenomenon” and data biases must be addressed to fully integrate AI into clinical practice. Zeppieri, Marsili et al. [26] complement this discussion with a review of OCT’s evolution and applications, highlighting its role in revolutionizing the diagnosis and management of retinal diseases. Together, these contributions underscore the transformative potential of technology in improving patient outcomes.

Emerging strategies for retinal disease management increasingly emphasize the importance of multimodal therapies that combine conventional and complementary interventions. Among these, integrative approaches—which synergistically target pathological pathways through pharmacological and nutraceutical means—have gained significant attention in recent years. This paradigm is exemplified by the work of Rusciano and Bagnoli [27], whose comprehensive review underscores the dual role of pharmacotherapy and nutritional supplements in managing neovascular retinal diseases. Their analysis demonstrates that this combined therapeutic strategy not only enhances treatment efficacy but also addresses key limitations of monotherapies, marking a significant advancement in retinal care. By integrating pharmacological and dietary interventions, such an approach aligns with modern paradigms of patient-centered medicine, optimizing outcomes through personalized, multimodal management. Retinopathy of prematurity (ROP) remains a leading cause of preventable childhood blindness worldwide, with incidence and severity closely tied to neonatal care standards and regional disparities. Recent studies underscore the critical need for early detection through systematic screening, particularly in populations with evolving healthcare infrastructure [28]. Contributing to this global effort, Simon-Szabo et al. [29] present a retrospective analysis of ROP in a Romanian cohort, elucidating modifiable risk factors—such as gestational age, oxygen therapy, and weight gain patterns—while demonstrating gaps in current screening practices. Their findings not only reinforce the universality of ROP’s risk profile but also deliver an urgent call for standardized protocols tailored to high-risk populations, offering actionable insights to reduce avoidable vision loss.

In conclusion, the 11 papers in this Special Issue collectively represent significant advancements in diagnosing, treating, and managing retinopathies. Innovative therapies such as PRGF and Faricimab, alongside the application of AI and genetic insights, highlight the critical need for a multidisciplinary approach in tackling the challenges posed by retinal diseases. By integrating clinical, genetic, and technological perspectives, this Special Issue enhances our understanding of retinopathies and lays the groundwork for future innovations aimed at improving patient outcomes. It serves as a valuable resource for researchers and clinicians, providing new insights and fostering ongoing progress in the field of ophthalmology.
